# Thoracic malignant schwannomma in adults

**DOI:** 10.4103/0970-2113.63617

**Published:** 2010

**Authors:** G. S. Gaude, P. R. Malur, R. Kangale, V. Dhorigol, S. Anurshetru

**Affiliations:** *Department of Pulmonary Medicine, J. N. Medical College, Belgaum - 590 010, Karnataka, India*; 1 *Department of Pathology, J. N. Medical College, Belgaum - 590 010, Karnataka, India*; 2 *Department of Cardiothoracic Surgery, J. N. Medical College, Belgaum - 590 010, Karnataka, India*

**Keywords:** Malignant schwannomma, malignant tumors of the nerve sheath origin, tumor, thoracic

## Abstract

A rare case of thoracic malignant schwannomma, in an adult, is presented here. This case shows an aggressive, rapid progression, which is characteristic of the disease. In spite of the best surgical and chemotherapy treatment, the patient died within four months of diagnosis.

## INTRODUCTION

Schwannomas (or neurilemommas) and neurofibromas are the most common mediastinal tumors. They are benign, slow-growing neoplasms that frequently arise from a spinal nerve root but may involve any thoracic nerve.[1] Malignant tumors of the nerve sheath origin (MTNSO) are rare spindle cell sarcomas. They represent the malignant counterparts of benign schwannomas and neurofibromas and have also been termed malignant neurofibromas, malignant schwannomas, and neurogenic fibrosarcomas.[2] We report a case of malignant schwannomas in an adult who showed an aggressive behavior pattern.

## CASE REPORT

**Figure 1:**
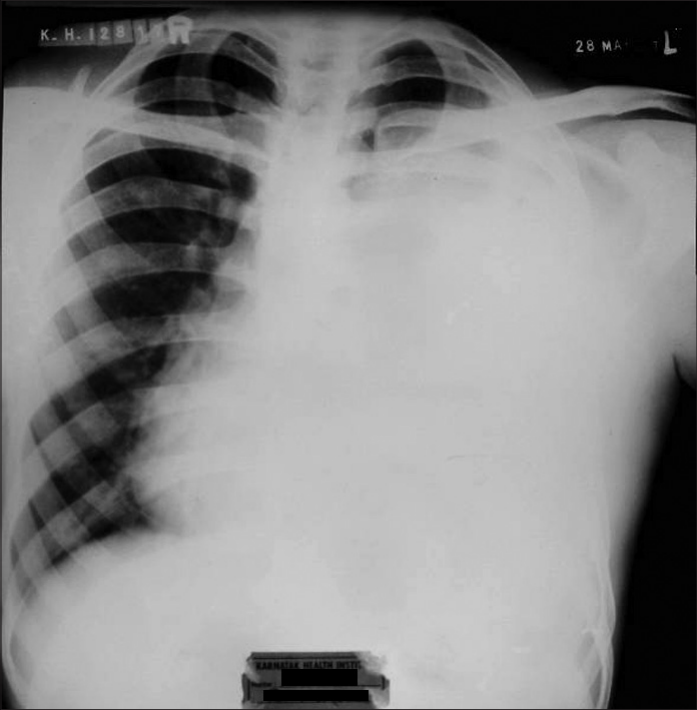
Chest radiograph showing large homogenous density occupying left hemi-thorax with obliteration of the left heart border and mediastinal shift to the right side

32-year-old male presented with chest pain and mild dyspnea of 20 days duration. He was a laborer and non-smoker with no history of exposure to any occupational or inorganic dust. Chest pain was retrosternal and pricking in character with no radiation and not related to the meals. Examination revealed averagely built person with BMI of 22, and no clubbing and lymphadenopathy. Respiratory system examination revealed diminished movements on the left hemi-thorax. There was stony dullness on the left hemi-thorax, and breath sounds were absent. No adventitious sounds were heard. Chest radiograph revealed a uniform density homogenous opacity in the left hemi-thorax occupying the full hemi-thorax except at the left apex. The mediastinum was shifted to the right side [Figure 1].

**Figure 2:**
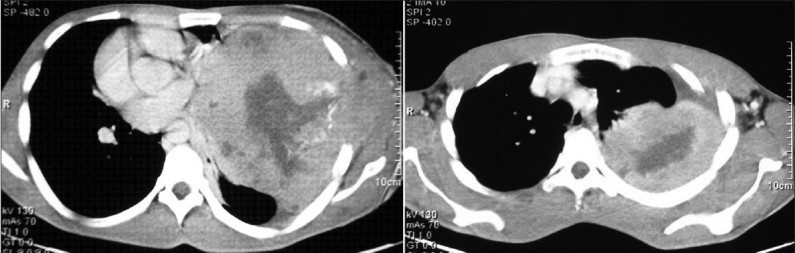
CT scan of the thorax showing large well defined heterogenous mass in the left hemi-thorax. The mass extends peripherally up to the pleura and chest wall, and shows infiltrations into the subcutaneous tissue. The mass also contains areas of low attenuation, corresponding to hemorrhage and necrosis, and extends up to the pleura and the chest wall. There is mediastinal shift to right side

Routine blood investigations were within normal limits. A Computed tomography (CT) of the thorax revealed a large well defined heterogenous mediastinal mass in the left hemi-thorax. There were some areas of necrosis and calcification within the mass. The mass was extending up to the pleural surface, and infiltrating into the subcutaneous tissues in the chest wall [Figure 2]. The diagnoses considered were anterior mediastinal tumours, posterior mediastinal tumours, pleural mesothelioma and bronchogenic carcinoma. Fibre-optic bronchoscopy revealed extrinsic compression of left main bronchus with no intra bronchial extension. Bronchial washings, bronchial brush biopsy and trans-bronchial needle aspiration (TBNA) of the mass were inconclusive. Trans-thoracic fine needle aspiration biopsy of the mass revealed small round tumor cells with thin strands of fibrous connective tissue.

**Figure 3:**
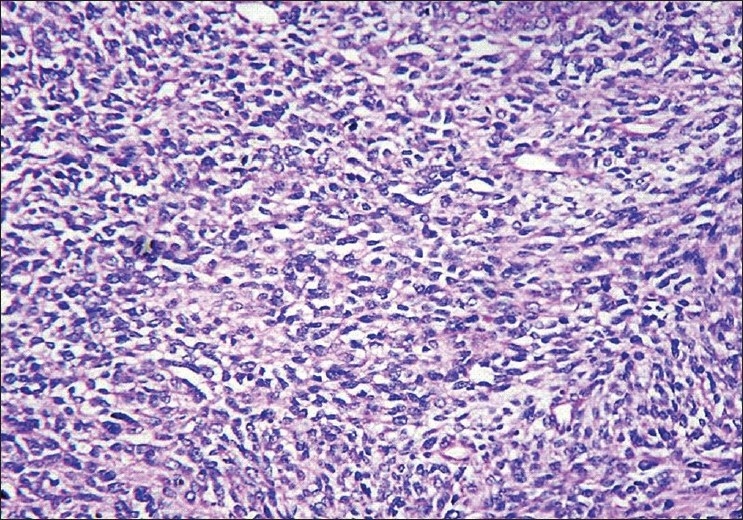
Biopsy specimen of the tumor showing malignant spindle cells. These tumor cells are arranged in fascicles (H and E, x400)

As the diagnosis was inconclusive, the patient was taken up for thoracotomy with excision of the tumor. Intra-operatively it was observed that there was a large firm mass in the mediastinum on the left side with invasion of the great vessels and involvement of mediastinal lymph nodes. The lymph nodes involved were: left hilar group, subcarinal group, aorto-pulmonary window group, right hilar group and left interlobar group of lymph nodes. The mass was seen infiltrating the great vessels, pericardium and the chest wall. Hence, de-bulking of the tumor was done to the extent possible. Complete excision of the tumor could not be done as it was infiltrating the great vessels and the pericardium. Histologically, the specimen revealed sheets of pleomorphic spindle shaped cells, with enlarged hyperchromatic twisted nuclei and amphophilic moderate eosinophilic cytoplasm. The neoplastic cells were arranged in fascicles that closely resembled the architecture of leiomyosarcomas [Figure 3]. They had numerous mitotic Figures and areas of necrosis. Another characterstic feature of malignant schwannomma observed was its ability to express other cellular components such as clusters of epithelial cells, and mucin-secreting glands. Immuno-histochemistry of the specimen proved the diagnosis of malignant schwannoma with tumor cells expressing S-100 protein, cytokeratin focal positivity, and desmin focal positivity. As the diagnosis of malignant tumor of nerve sheath origin was established and wide surgical excision of the tumor already being done, he was referred for radiotherapy and chemotherapy. The patient received radiotherapy with 4000 Rads and three cycles of chemotherapy with Cisplatinum, Bleomycin and Doxorubicin. The patient continued to be symptomatic and his condition worsened over a period of the next three months. He died, subsequently, four months after the diagnosis.

## DISCUSSION

Neurogenic tumor is the most common cause of a mass lesion in the thoracic para-vertebral region in the general adult population. Schwannomas are rare in people less than 20 years of age and are largely asymptomatic.[3] Schwannomas arise from the nerve sheath and extrinsically compress the nerve fibers. They are often found in individuals with neurofibromatosis or von Recklinghausen disease. They are encapsulated neoplasms composed of Schwann cells within a background of loose reticular tissue without nerve fibrils or collagen.[4] Frequently heterogenous, especially when large, they show areas of cystic degeneration, low cellularity, hemorrhage, myelin, and small calcification.

Malignant schwannomas typically arise from a simple or plexiform neurofibroma,[5] but rarely, if ever, from a pre-existing schwannoma. They are extremely pleomorphic and cellular malignancies. They affect men and women in the third through fifth decade.

Approximately half of the cases occur in individuals with neurofibromatosis and the incidence of sarcomatous degeneration is seen in five per cent cases. Malignant schwannomas may occur sporadically or be induced by radiation exposure.[6] Most of the patients with malignant schwannomas are adolescents or young adults. Chest pain, an enlarging mass, and/or symptoms of nerve deficit are the common presentations, and may persist for several months or years, and are frequently associated with a long standing mass. Local recurrence is quite common. In this case symptoms lasted for 20 days. It could be negligence of symptoms by the patient due to financial constraints.

Differential diagnosis of the dense homogenous density lesion in the left hemithorax, similar to the present case, includes bronchogenic carcinoma, lymphomas (Hodgkins or non-Hodgkins disease), pleural mesothelioma, thymic tumours, teratomas, and tumours arising from sympathetic ganglia like ganglioneuroma, ganglioneuroblastoma and neuroblastomas, and encysted pleural effusion. Radiologically, schwannomas appear as a well circumscribed, round masses which are homogenous, soft-tissue density on plain CT images, with clear preservation of surrounding fat planes.[7] Occasionally they may be seen as areas of very low attenuation, on non-contrast CT examinations, if there is a high concentration of lipid-rich Schwann cells in these tumors. Malignant schwannomas manifest as spherical posterior mediastinal masses, which usually exceed five cms in diameter. Malignant schwannomas have CT findings of low density areas, compression of adjacent structures, pleural abnormalities, such as pleural effusion or pleural nodules, and metastatic pulmonary nodules.[3] Areas of low attenuation correspond to central hemorrhage and necrosis. Calcification may be present. On MRI, schwannomas show low-to-intermediate signal density on T1–weighted images, while on T2-weighted images, they show in-homogenously high density. Very high intensity region seen on T2-weighted images of schwannomas correspond to cystic degeneration with surrounding collagen fibrous tissue.[3] Majority of the times either tru-cut biopsy of the mass or VATS will be required to prove the diagnosis, especially in mediastinal tumours,[8] as other procedures including bronchoscopy fails to give the definite diagnosis. Hematogenous metastases, most frequently to the lungs, are common in malignant schwannomas but lymph node metastases are rare. Radical surgical excision with wide margins is the procedure of choice.[6] Simple excision without wide margins or subtotal excision followed by high dose radiation therapy is an alternative when complete resection is not possible. Adjunct radiation therapy and chemotherapy do not appear to improve survival, but may have utility in the treatment of metastatic diseases. No known chemotherapeutic regimens are effective against these tumours. Local recurrence following incomplete excision is frequent. The overall survival is poor and is adversely affected by large tumor size, incomplete resection, and/or an association with neurofibromatosis.[6] Recurrences and metastasis are more common in patients with neurofibromatosis.

In this case, diagnosis could not be reached following routine procedures, and hence exploratory thoracotomy was performed. It confirmed malignant schwannomma. In this case, wide excision of the tumor was done. Complete excision was not possible due to the invasion of the great vessels and chest wall. He was also given adjunct radiation therapy and chemotherapy. However, due to rapid progression of the tumor, the patient succumbed to the disease within four months of diagnosis.

## References

[1] Aughebough GL (1984). Thoracic manifestations of neurocutaneous diseases. Radiol Clin North Am.

[2] Ribert ME, Cardot GR (1994). Neurogenic tumors of the thorax. Ann Thorac Surg.

[3] Strub WM, Leach JL, Wand A (2005). Thoracic schwannoma. Appl Radio.

[4] Kumar AJ, Kuhajda FP, Martinez CR (1983). Computed tomography of the cranial nerve sheath tumors with pathological correlation. J Comput Assist Tomogr.

[5] Ghosh BC, Ghosh L, Huvos AG, Fortner JG (1973). Malignant schwannoma. Cancer.

[6] Strollo DC, Rsado-de-Christenson ML, Jett JR (1997). Primary mediastinal tumors: Part II.Tomors of the middle and posterior mediastinum. Chest.

[7] Powers SK, Norman D, Edwards MS (1983). Computed tomography of peripheral nerve lesions. J Neurosurg.

[8] Ducatman BS, Scheithauer BW, Piepgras DG, Reiman HM, Ilstrup DM (1986). Malignant peripheral nerve sheath tumors: A clinicopathologic study of 120 cases. Cancer.

